# Impact of a Three-Week in-Hospital Multidisciplinary Body Weight Reduction Program on Body Composition, Muscle Performance and Fatigue in a Pediatric Obese Population with or without Metabolic Syndrome

**DOI:** 10.3390/nu12010208

**Published:** 2020-01-13

**Authors:** Antonello Emilio Rigamonti, Gabriella Tringali, Roberta De Micheli, Alessandra De Col, Sofia Tamini, Antonella Saezza, Silvano G. Cella, Alessandro Sartorio

**Affiliations:** 1 Department of Clinical Sciences and Community Health, University of Milan, 20129 Milan, Italy; silvano.cella@unimi.it; 2 Experimental Laboratory for Auxo-Endocrinological Research, Istituto Auxologico Italiano, IRCCS, 28824 Piancavallo (VB), Italy; gabriella.tringali@yahoo.it (G.T.); roby88demicheli@gmail.com (R.D.M.); a.decol@auxologico.it (A.D.C.); sofia.tamini@gmail.com (S.T.); sartorio@auxologico.it (A.S.); 3 Division of Auxology and Metabolic Diseases, Istituto Auxologico Italiano, IRCCS, 28824 Piancavallo (VB), Italy; a.saezza@auxologico.it

**Keywords:** metabolic rehabilitation, body weight reduction program, diet, adapted physical activity, psychological counselling, pediatric obesity, metabolic syndrome

## Abstract

Metabolic syndrome is a combination of cardiometabolic risk factors, frequently detected in obese children and adolescents. To date, few clinical studies have evaluated the effectiveness of multidisciplinary body weight reduction programs on body mass index, body composition, muscle performance and fatigue in pediatric obese subjects suffering from metabolic syndrome, which might represent a sub-population that is more difficult to be treated and worthy of more intensive interventions than a population less metabolically complicated. The aim of the present study was to compare the impact of a three-week in-hospital multidisciplinary integrated body weight reduction program (BWRP) on body mass index (BMI), body composition (particularly, fat mass (FM) and fat-free mass (FFM)), motor control (evaluated by one-leg standing balance (OLSB) test), muscle performance (evaluated by the stair climbing test (SCT)) and fatigue (evaluated by fatigue severity scale (FSS)) in a pediatric obese population with or without metabolic syndrome. A pediatric population of 548 obese subjects without metabolic syndrome (F/M = 312/236; age range: 8–18 years; BMI: 36.3 ± 6.7 kg/m^2^) and 96 obese subjects with metabolic syndrome (F/M = 53/43; age range: 9–18 years; BMI: 38.3 ± 6.9 kg/m^2^) was recruited. The BWRP significantly reduced BMI, FM (expressed as %), SCT time and FSS score, and increased OLSB time in all subgroups of obese subjects, independent of sex and metabolic syndrome, with preservation of FFM. No significant differences in |ΔBMI|, |ΔFM|, |ΔOLSB| or |ΔSCT| times and |ΔFSS| score were found when comparing subjects (males and females) with or without metabolic syndrome, apart from obese females without metabolic syndrome, who exhibited a lower weight loss and FM (expressed as %) reduction when compared to the corresponding male counterpart. In conclusion, the beneficial effects of a three-week BWRP on BMI, body composition, muscle performance and fatigue in a pediatric obese population were not found to be different in patients with or without metabolic syndrome, thus indicating that the more metabolically compromised patient is as responsive to a short-term BWRP as the patient without metabolic syndrome. More prolonged follow-up studies are, however, necessary in order to verify whether the adherence to the multidisciplinary recommendations at home and the long-term maintenance of the positive effects in the two subgroups of patients will remain similar or not.

## 1. Introduction

Pediatric obesity represents an increasingly serious problem of public health in both developed and developing countries [1,2]. Metabolic syndrome is a combination of different cardiometabolic risk factors, including abdominal adiposity, hypertension, dyslipidemia and hyperglycemia, associated with atherosclerosis, cardiovascular diseases, non-alcoholic fatty liver disease, type 2 diabetes mellitus and cerebrovascular diseases [3]. This nosographic picture, previously diagnosed uniquely in obese adults, is now frequently recognized in obese children and adolescents, for whom specific diagnostic criteria have been elaborated [4,5].

The most alarming finding, which is derived from a long-term forthcoming study, is the 15-fold increase in the risk of premature cardiovascular disease among adults that were diagnosed to have a metabolic syndrome in childhood [6]. Therefore, early adoption of large-scale healthy lifestyle changes and therapeutic interventions, including multidisciplinary integrated body weight reduction programs (BWRP), is fundamental for controlling the pandemic problem of pediatric obesity and, more importantly, to arrest the progression of metabolic syndrome [7].

While the short- and long-term effectiveness of a BWRP in obese children and adolescents has been demonstrated by several clinical studies [8], to the best of our knowledge, few authors have investigated the effects of diet and/or exercise in pediatric metabolic syndrome. As severely obese subjects are generally more difficult to treat through a standardized BWRP [9], one might argue that an obese subject with metabolic syndrome will respond less to a BWRP (or other therapeutic interventions) when compared to an obese subject without metabolic syndrome.

The potential benefits associated with weight loss in an obese subject should be established by measuring change in validated outcomes following the participation in any BWRP. Outcomes either directly measure the change of an adiposity-related clinical parameter (so-called primary outcomes such as weight and body composition) or assess the change of a surrogate (not necessarily clinical) parameter that impacts/depends on the primary outcome (so-called secondary outcomes such as time spent watching television). In the design of any clinical study in pediatric obesity, choosing appropriate outcomes is essential to demonstrate the effectiveness of any specific short- and long-term BWRP administered to obese children and adolescents. In this respect, several outcomes have been proposed, including anthropometry, diet, eating behaviors, physical activity, sedentary time/behavior, fitness, physiology, biochemistry, environment, psychological well-being and health-related quality of life [10,11].

In particular, outcomes related to physical activity have the advantage of being measured with a high degree of precision and are often feasible to obtain from clinical practice. These characteristics can be found in three tests, which, in the last years, our group has standardized and validated in obese subjects: (1) one-leg standing balance (OLSB) test, a simple quantitative method to assess static postural control and balance control; (2) stair climbing test (SCT), used to evaluate functional strength, balance and agility of lower limbs through ascending a set number of steps; and (3) fatigue severity scale (FSS), a largely employed self-report questionnaires to evaluate fatigue in daily activities, which does not depend upon an underlying depressive condition.

The aim of the present study, therefore, was to evaluate the effects of a three-week BWRP on weight, body composition, motor control (by OLSB time), muscle performance (by SCT time) and fatigue (by FSS score) in a large pediatric obese population with or without metabolic syndrome. Our hypothesis is that, when obese children and adolescents undergo a multidisciplinary intervention of metabolic rehabilitation, the ensuing response is similar between the groups with or without metabolic syndrome.

## 2. Material and Methods

### 2.1. Patients and Body Weight Reduction Program

From January 2016 to September 2019, a pediatric population of 595 obese subjects without metabolic syndrome (F/M = 340/255) and 109 obese subjects with metabolic syndrome (F/M = 61/48) was selected at the Division of Auxology, Istituto Auxologico Italiano, Piancavallo (VB), where they were hospitalized for a three-week multidisciplinary integrated BWRP, entailing hypocaloric diet, nutritional education, psychological counselling and moderate physical activity (see below for details). Because of declining to participate (expressed by the patients and/or their parents), medical reasons (i.e., physical inability or cognitive impairments hampering the execution of the tests) and a refusal to complete the three-week hospitalization period (self-discharge for personal reasons) (*n* = 60; F/M = 36/24, i.e., 28/19 without metabolic syndrome and 8/5 with metabolic syndrome), 548 obese subjects without metabolic syndrome (F/M = 312/236; age range: 8–18 years; body mass index, BMI: 36.3 ± 6.7 kg/m^2^) and 96 obese subjects with metabolic syndrome (F/M = 53/43; age range: 9–18 years; BMI: 38.3 ± 6.9 kg/m^2^) were recruited.

The multidisciplinary integrated BWRP (on average 25 days), adopted in the present study, was allowed and refunded by the Italian National Health Service for severe childhood obesity. 

The unique criterion of inclusion was a BMI > 95th percentile from age- and sex-specific Italian charts [12], while the main exclusion criteria were physical inability or cognitive impairments hampering the execution of the tests included in the present study (see below for a detailed description).

As far as the energy intake component of the BWRP is concerned, the subjects consumed a diet (5023–7113 kJ/day, i.e., 1200–1700 kcal/day) containing about 21% protein, 53% carbohydrates and 26% lipids. The calories to be given with diet were calculated by subtracting approximately 25% from the value of resting energy expenditure as measured in each patient by indirect calorimetry (V_max_ 29; SensorMedics Corporation, Yorba Linda, CA, USA) for a total duration of 20 min. Under the energy restriction period, each patient was free to choose foods from a heterogeneous daily menu. Foods which the patient was declared to be allergic to were removed from his/her menu. Five daily portions of fruits and vegetables were obligatory. A fluid intake of at least 1500 mL/day was recommended. During the whole BWRP, nutritional education was planned every day, consisting of lectures, demonstrations and group discussions with and without a supervisor. Clinical psychologists performed sessions of psychological counselling 2–3 times/week, based on individual or cognitive behavioral strategies. Physical activity consisted of five training sessions/week (about 1 h/session), including indoor light jogging, dynamic exercises of the upper and lower limbs (standing and floor gymnastics routines, aimed at muscle strength–power development) at moderate intensity under the guide of qualified trainers. The scheduled physical activity was further complemented with either 15–20 min aerobic exercise or 2 km outdoor walking on a predetermined track, according to individual capabilities and clinical status.

Before and after BWRP, each subject underwent the following tests/evaluations, which are described in detail below:

- OLSB (one-leg standing balance);
- SCT (stair climbing test);
- FSS (fatigue severity scale).

The protocol was approved by the local Ethical Committee (research project code: 01C824; acronym: POTARTINFOB); all subjects or their parents gave their written consent after having been fully informed of the nature and procedures of the study.

The datasets used and/or analyzed in the present study are available from the corresponding author on reasonable request.

### 2.2. Anthropometric Measurements

A scale with a stadiometer was used to determine the height and the weight of the individuals (Wunder Sa.Bi., WU150, Trezzo sull’Adda, Italy). Waist circumference was measured with a flexible tape in standing position, halfway between the inferior margin of the ribs and the superior border of the crista, while hip circumference was measured as the greatest circumference around the nates. Body composition was measured by bioimpedance analysis (Human-IM Scan, DS-Medigroup, Milan, Italy) after 20 min of supine resting and in accordance with international guidelines [13].

### 2.3. Metabolic Variables

Blood samples (a total of about 20 mL) were collected at around 8:00 AM after an overnight fast only at the beginning of the BWRP. Lipids (high-density lipoprotein (HDL) cholesterol and triglycerides) and glucose were measured. 

Serum glucose level was measured by the glucose oxidase enzymatic method (Roche Diagnostics, Monza, Italy). The sensitivity of the method was 2 mg/dL [1 mg/dL = 0.06 mmol/L].

Colorimetric enzymatic-assays (Roche Diagnostics, Monza, Italy) were used to determine serum HDL cholesterol and triglycerides levels. The sensitivities of the assays were 3.09 mg/dL [1 mg/dL = 0.03 mmol/L] and 8.85 mg/dL [1 mg/dL = 0.01 mmol/L], respectively.

### 2.4. Evaluation of Blood Pressure

Blood pressure (BP) was measured on the right arm, using a sphygmomanometer with pediatric cuff size, with the subject in a seated position and relaxed condition. The procedure was repeated three times at 10 min intervals in-between; the means of the three values for systolic BP (SBP) and diastolic BP (DBP) were recorded.

### 2.5. Definition of Metabolic Syndrome

According to the IDF (International Diabetes Federation) criteria for diagnosis of metabolic syndrome in children and adolescents [14], our patients were considered positive for the presence of metabolic syndrome if they had abdominal obesity (waist circumference [WC] ≥ 90th percentile [15] for ages <16 years, and ≥94 cm for males and ≥80 cm for female for ages >16 years) plus two or more of the following factors: (i) increased triglycerides level: ≥150 mg/dL (1.7 mmol/L) for ages <16 years and the same cut-off or specific treatment for this lipid abnormality for ages >16 years, (ii) reduced HDL cholesterol: <40 mg/dL (1.03 mmol/L) for males and females for ages <16 years, and <40 mg/dL for males and <50 mg/dL (1.29 mmol/L) for females, or specific treatment for this lipid abnormality for ages >16 years, (iii) increased BP: SBP ≥ 130 mmHg or DBP ≥ 85 mmHg for ages <16 years, and same cut-off or treatment of previously diagnosed hypertension for ages >16 years, (iv) increased fasting plasma glucose (FPG) concentration ≥100 mg/dL (5.6 mmol/L) or previously diagnosed type 2 diabetes mellitus for all ages.

### 2.6. Functional Tests

The following functional tests were performed randomly at the beginning and repeated at the end of the BWRP period (i.e., after 21 days):

(1) One-leg standing balance (OLSB). The subjects were invited to stand on one leg with the other flexed for as long as possible, looking straight ahead. The test was considered to be terminated with the ground contact of the flexed leg or with an overt loss of equilibrium, although compensatory movements of arms and lifted leg were allowed. An operator registered the value in seconds with a digital stopwatch. The test was repeated for both legs (right and left) in order to obtain two OLSB values (i.e., OLSB_R_ and OLSB_L_).
(2) Stair climbing test (SCT). The subjects were invited to climb up ordinary stairs (13 steps of 15.3 cm each, for a total vertical distance of 1.99 m) at the highest possible speed, according with their own capabilities. An operator measured the time employed to cover the test with a digital stopwatch. The test was considered to start at the moment when the first foot was lifted and to terminate with the contact of the same foot on the last step.

At the moment of the first execution of both tests (i.e., basal condition), 2–3 practice trials were allowed so that the subject gained a good control of the performing technique. No further repetition of both tests was allowed during the three-week BWRP period.

### 2.7. Fatigue Severity Scale

FSS is one of the most commonly used self-report questionnaires for fatigue assessment in chronic diseases [16,17], and was already used and validated in Italian obese patients studied by our group [18,19].

FSS consists of nine statements (items) describing the negative effects of fatigue on motivation, exercise, physical functioning, ability to carry out duties, work, family or social life. The subject was asked to rate each statement considering the previous week, using a Likert scale ranging from 1 (strong disagreement) to 7 (strong agreement). The total score was computed by averaging the raw scores of each item.

### 2.8. Statistical Analysis

The Sigma Stat 3.5 statistical software package (Systat Software, San Jose, CA, USA) was used for data analyses and GraphPad Prisma 5.0 software (GraphPad Software, San Diego, CA, USA) for data plotting.

Results are reported as mean ± SD (standard deviation). Each of the parameters, particularly BMI, FM (kg and %), FFM, FSS, OLSB and SCT, were evaluated not only as continuous variables, but also as pre–post-BWRP difference in absolute value (|Δ| in the corresponding unit of measurement for all parameters). In particular, if the pre–post-BWRP difference itself was negative (Δ < 0), then the corresponding positive value was considered (|Δ| = −Δ > 0).

All parameters were compared among all subgroups (all, females/males, obese with/without metabolic syndrome) before and/or after BWRP by using a Wilcoxon signed-rank test or a Kruskal–Wallis one-way ANOVA on ranks, when appropriate.

A level of significance of *p* < 0.05 was used for all data analyses.

## 3. Results

### 3.1. Patients’ Parameters

Demographic, clinical and biochemical parameters of the recruited pediatric obese population are reported in Table 1, which includes the statistical significance of the possible comparisons among groups/subgroups. In particular, weight, BMI, waist, waist to hip ratio (WHR), glucose, triglycerides, heart rate (HR) and SBP were significantly lower in obese subjects without metabolic syndrome than those with metabolic syndrome (*p* < 0.05). On the contrary, HDL-cholesterol was higher in the obese group without metabolic syndrome than in patients with metabolic syndrome (*p* < 0.05). Obese females without metabolic syndrome had significantly lower values of weight, waist, WHR, triglycerides and SBP than obese females or males with metabolic syndrome (*p* < 0.05), with HDL-cholesterol being significantly higher (*p* < 0.05). Weight, waist and WHR were significantly lower in obese females without metabolic syndrome than the corresponding male subgroup (*p* < 0.05). Glucose was significantly lower in obese females without metabolic syndrome than obese males with metabolic syndrome (*p* < 0.05). Triglycerides were significantly lower in obese males without metabolic syndrome than females and males with metabolic syndrome, with HDL-cholesterol being significantly lower (*p* < 0.05). Obese males without metabolic syndrome had significantly lower HR than obese females with metabolic syndrome (*p* < 0.05).

### 3.2. BMI and Body Composition

Before BWRP, obese subjects with metabolic syndrome had a significantly higher BMI than those without metabolic syndrome (*p* < 0.05), a difference that disappeared when considering the sex-specific subgroups. BWRP significantly reduced BMI in all subgroups, independent of sex or metabolic syndrome. Despite this weight loss, BMI in obese subjects with metabolic syndrome remained significantly higher than in those without metabolic syndrome (*p* < 0.05) (Table 2).

As expected, before BWRP, FM expressed as % was significantly higher in females than males with or without metabolic syndrome (*p* < 0.05), a difference that was not observed when FM was expressed in kg. FM expressed as % was significantly reduced by the BWRP in all subgroups, including the subjects with metabolic syndrome of both sexes (*p* < 0.05). Similar results were observed when FM was expressed in kg (*p* < 0.05), with the exception of the sex-specific subgroups with metabolic syndrome (Table 2).

As expected, before BWRP, FFM was significantly lower in females than males with or without metabolic syndrome (*p* < 0.05), a difference that, as with FM expressed in %, significantly persisted after BWRP (*p* < 0.05) (Table 2).

BWRP failed to significantly change FFM, indicating a preservation of muscle mass (Table 2). 

**Figure 1:**
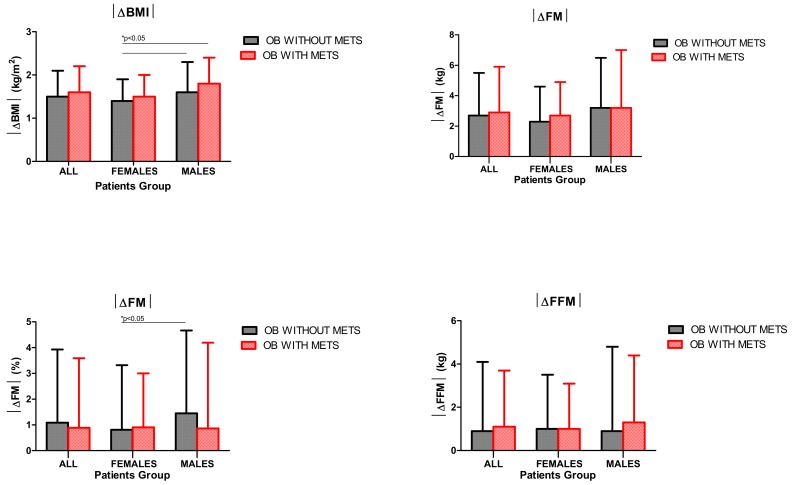
Absolute values of changes (|Δ|) of body mass index (|ΔBMI|, top left panel), fat mass (|ΔFM|) expressed as kg (top right panel), |ΔFM| expressed as % (bottom left panel) and fat-free mass (|ΔFFM|, bottom right panel), before and after a three-week body weight reduction program (BWRP) in obese (OB) children and adolescents with or without metabolic syndrome (METS). Data are expressed as mean ± SD. The values corresponding to each sex-related subgroup (females/males) are reported. All parameters were compared among all subgroups (all, females/males, obese with/without metabolic syndrome) before and/or after the body weight reduction program (BWRP) by using a Wilcoxon signed-rank test (two groups) or a Kruskal–Wallis one-way ANOVA on ranks (more than two groups), when appropriate.

When comparing the different subgroups of obese subjects in terms of |ΔBMI|, |ΔFM| and |ΔFFM|, no significant differences were found, apart from obese females without metabolic syndrome, who appeared to lose less weight (|ΔBMI|) and FM (|ΔFM| expressed in %) when compared with the corresponding male counterpart (*p* < 0.05). Moreover, weight loss in obese females without metabolic syndrome was lower than in obese males with metabolic syndrome (*p* < 0.05) (Figure 1).

### 3.3. OLSB

Before BWRP, no significant differences in OLSB_R_ and OLSB_L_ times were observed when comparing all subgroups (females/males and with/without metabolic syndrome). Importantly, BWRP significantly increased OLSB_R_ and OLSB_L_ times in all subgroups, including those with metabolic syndrome (*p* < 0.05) (Table 2).

**Figure 2:**
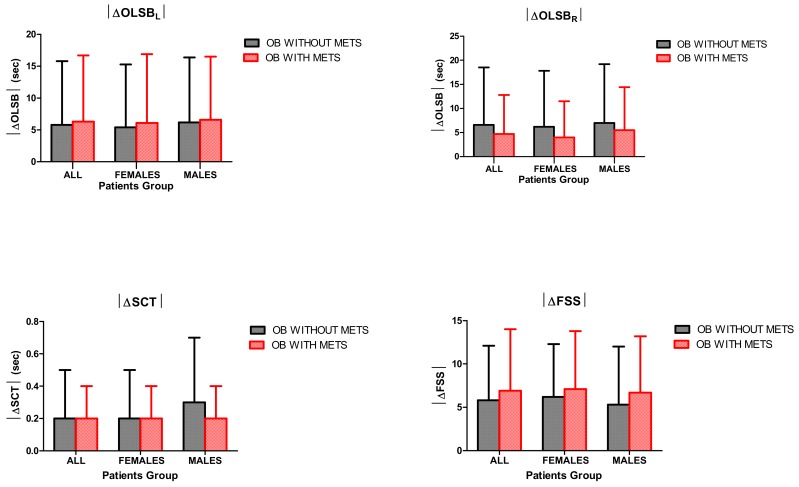
Absolute values of changes (|Δ|) of changes of one-leg standing balance (for the left side, |ΔOLSB_L_|, top left panel), |ΔOLSB_R_| (for the right side, top right panel), stair climbing test (|ΔSCT|) time (bottom right panel) and fatigue severity scale (|ΔFSS|) score (bottom left panel), before and after a three-week body weight reduction program (BWRP) in obese (OB) children and adolescents with or without metabolic syndrome (METS). Data are expressed as mean ± SD. The values corresponding to each sex-related subgroup (females/males) are reported. All parameters were compared among all subgroups (all, females/males, obese with/without metabolic syndrome) before and/or after BWRP by using a Wilcoxon signed-rank test (two groups) or a Kruskal–Wallis one-way ANOVA on ranks (more than two groups), when appropriate.

When comparing the different subgroups of obese subjects in terms of |ΔOLSB_R_| and |ΔOLSB_L_| times, no significant differences were found, indicating that the beneficial effect of BWRP on motor control is similar in obese subjects, independent of from sex and metabolic syndrome (Figure 2).

### 3.4. SCT

As expected, before BWRP, SCT time was significantly higher in obese females than males with or without metabolic syndrome (*p* < 0.05). Nevertheless, no significant difference in SCT time was observed when comparing all obese subjects with vs. without metabolic syndrome. Importantly, BWRP significantly reduced SCT time in all subgroups, including those with metabolic syndrome (*p* < 0.05) (Table 2).

When comparing the different subgroups of obese subjects in terms of |ΔSCT| time, no significant differences were found, indicating that the beneficial effect of BWRP on muscle performance is similar in obese subjects independent of sex and metabolic syndrome (Figure 2).

### 3.5. FSS

Before BWRP, no significant differences in FSS score were observed when comparing all subgroups (females/males and with/without metabolic syndrome). Importantly, BWRP significantly reduced FSS score in all subgroups, including those with metabolic syndrome (*p* < 0.05) (Table 2).

When comparing the different subgroups of obese subjects in terms of |ΔFSS| score, no significant differences were found, indicating that the beneficial effect of BWRP on fatigue is similar in obese subjects independent of sex and metabolic syndrome (Figure 2).

## 4. Discussion

To the best of our knowledge, for the first time, the effectiveness of a three-week BWRP, entailing restricted energy intake, moderate aerobic exercise, nutritional education and psychological counselling, was compared between two groups of obese children/adolescents (i.e., with and without metabolic syndrome) by evaluating specific outcomes of auxometry, body composition, motor control, muscle performance and psychological well-being.

The main findings of the present study, carried out in a large pediatric population of obese females and males with or without metabolic syndrome, who had undergone a three-week in-hospital BWRP, are the following:

(1) BWRP significantly reduced BMI and FM (expressed as %) in all subgroups of obese subjects (females/males and with/without metabolic syndrome), with preservation of FFM;
(2) BWRP significantly improved motor control, muscle performance and fatigue perception in all subgroups of obese subjects (females/males and with/without metabolic syndrome);
(3) the pre–post-BWRP change of each outcome (i.e., |ΔBMI|, |ΔFM|, |ΔOLSB|, |ΔSCT| and |ΔFSS|) was similar between the two obese groups with and without metabolic syndrome;
(4) obese females without metabolic syndrome reached a lower weight loss after BWRP when compared to obese males with and without metabolic syndrome, as demonstrated by the significantly lower values of |ΔBMI| and |ΔFM| (expressed as %).

Simple, economic, accurate and reproducible indicators of physical and psychological health among obese patients are of great interest for the promotion of lifestyle changes, development of new therapeutic interventions, guidance of health policy and supply of clinical services for obese population, particularly the geriatric and pediatric ones. 

OLSB is a useful clinical indicator of motor control, resulting from complex physiologic central and peripheral processes, which, in obese individuals (due to excessive body mass, sarcopenia and osteoarticular problems), is frequently impaired in comparison to the normal-weight counterpart [20,21]. In this respect, Sartorio et al. [22] have demonstrated that a three-week BWRP, similar to that administered to our pediatric population, was able to increase OLSB time in obese adults (aged 18–77 years). If, in a (normal-weight or obese) geriatric population, OLSB predicts the risk for injurious falls [23], then OLSB improvement in obese children and adolescents might be an objective and sensitive outcome that shows the beneficial effect of a BWRP on motor control, which is a fundamental requisite to sustaining a continuous physical activity in out-patient settings after in-hospital completion of a BWRP, particularly in severely obese adolescents with metabolic syndrome [24,25].

Similar considerations are also valid for SCT time, which, evaluating specifically muscle performance [26,27], has recently been reported to decrease after a three-week BWRP administered to geriatric and pediatric obese populations (with no indication regarding the state of metabolic syndrome) [18,28]. In this respect, Lazzer et al. [28] enrolled more than 3500 obese children and adolescents (aged 8–18 years) to be included in a BWRP and showed a sex- and age-dependent increase of the lower limb muscle power (LLMP), a surrogate endpoint derived from the measured SCT time. In the present study, due to the impossibility of forming age- and sex-specific subgroups of adequate size, the data were then pooled, impeding us to perform an analysis for sex and age. Anyway, as the BWRP, reportedly, failed to increase LLMP (in absolute value) within the subgroups of obese <13 years old girls and <12 years old boys [28], the beneficial effect of BWRP on SCT might be more relevant when considering adolescents separately from children, having the former reached a more advanced pubertal stage than the latter, including a higher ratio of FFM/FM, which would favor better muscle performance [29].

Fatigue is a symptom frequently complained of by obese patients, particularly those with a long duration of the disease or with advanced age [17,18,30]. Fatigue represents one of the most important reasons for failure, non-adherence or drop-out in any exercise-based BWRP in a pediatric obese population [31,32]. In the present study, FSS score decreased at the end of the BWRP in all subgroups of obese subjects (females/males and with/without metabolic syndrome), both confirming and extending the results of one of our previous studies, in which severely obese adults (age: 18–83 years and BMI: 35.0–65.3 kg/m^2^) underwent a similar intervention [19]. The relief from fatigue may be attributed to several reasons: restoration of a physical agility due to weight loss, improvement of some biochemical parameters with beneficial impact on muscle metabolism, or psychological well-being as a consequence of the well-known anti-depressive effect of exercise, which is a component of our BWRP [33].

Importantly, all of these BWRP-induced favorable effects on BMI, body composition (FM), motor control (OLSB time), muscle performance (SCT time) and fatigue (FSS score), were similar when comparing our obese children and adolescents with or without metabolic syndrome. Indeed, obese females with metabolic syndrome seemed to respond (apparently) better than the corresponding counterpart without metabolic syndrome in terms of |ΔBMI| and |ΔFM| (expressed as %). 

Obesity combined with metabolic syndrome has been reported to be less responsive to standard medical interventions such BWRP [9,34], which, when ineffective, have been proposed to be replaced by bariatric surgery [35]. The present study included a pediatric obese population, which, different to an adult one, appears to be less compromised by metabolic syndrome, which, in a longer time frame, causes multi-systemic damage of predominantly atherosclerotic origin [36]. Therefore, one might argue that the success of our BWRP on specific outcomes in obese children and adolescents with metabolic syndrome may rely on the “reversibility” of the metabolic-syndrome-induced alterations in the youth and that any therapeutic intervention against obesity and, particularly, metabolic syndrome, should be adopted as early as possible in childhood and adolescence [7]. This might have some implications when our public health stakeholders are called to make decisions to fight pediatric obesity [37,38,39,40,41,42].

Before closing, some limitations of our study should be mentioned. 

First, due to the short period (three weeks) between the two determinations (before and after BWRP), we have taken the decision of not measuring biochemical parameters at the end of the study, including those concurring with the definition of metabolic syndrome (i.e., glucose, triglycerides and HDL cholesterol). In this respect, as reported in one of our previous studies [43], three weeks were not sufficient to favorably change biochemical parameters in metabolic syndrome. A more prolonged period of BWRP is likely to be needed to demonstrate favorable changes in biochemical parameters related to the definition of metabolic syndrome.

Second, in the present study, we evaluated outcomes of physical activity only by means of specific tests (i.e., SCT, OLSB and FSS) that have been standardized and validated by our group in the obese population. In our opinion, it is difficult to compare these tests with those adopted in other studies in order to establish the effectiveness of any BWRP [10]. Head-to-head comparisons of these tests (the ours vs. the others) should be performed in future studies.

Furthermore, when considering the outcomes used in the present study (i.e., |ΔBMI|, |ΔFM|, |ΔFFM|, |ΔOLSB|, |ΔSCT| and |ΔFSS|), we were able to find some sex-related differences in the effectiveness of BWRP between obese females vs. males. In particular, |ΔBMI| and |ΔFM| (in %) were significantly lower in obese females without metabolic syndrome than the corresponding male group. While there are a number of systematic reviews that have investigated the effectiveness of different BWRPs with a focus on either men or women, or men and women combined [44,45,46,47,48], few authors have specifically studied the differences in weight loss between men and women, and of these, the results that have been reported have been conflicting [49]. There are some “opposing” physiological differences between males and females that may explain the sex-related variation in weight loss success (e.g., higher energy expenditure in men than women due to the favorable FFM/FM in the formers or higher levels of leptin in women than men with the favorable suppression of appetite in the formers) [50,51,52]. The differences in the administered BWRP seem to represent the most relevant factor that contributes to weight loss success in men or women [49].

Lastly, our BWRP consists of a combination of hypocaloric diet, moderate aerobic activity, nutritional education and psychological counselling; therefore, we are unable to know the effects of a diet-alone- or exercise-alone-based BWRP on SCT, OLSB and FSS to date. It seems plausible to hypothesize that the effects exerted by an unimodal BWRP are lower, seeing as though the multimodal intervention is usually more effective in reducing BMI and improving body composition [53,54].

In conclusion, the beneficial effects of a three-week BWRP on BMI, body composition, motor control, muscle performance and fatigue in a pediatric obese population are not different in patients with or without metabolic syndrome, thus indicating that the more metabolically compromised patient is equally (positively) responsive to a short-term BWRP as the patient without metabolic syndrome. More prolonged follow-up studies are, however, necessary in order to verify whether the adherence to the multidisciplinary recommendations at home and the long-term maintenance of the positive effects in the two subgroups of patients will remain similar or not.

## Figures and Tables

**Table 1:** Demographic, clinical and biochemical parameters of the recruited pediatric population, subdivided for metabolic syndrome (with/without) and sex (female/male).

Parameter	Obese without Metabolic Syndrome			Obese with Metabolic Syndrome		
	All	Females	Males	All	Females	Males
N.	548	312	236	96	53	43
Age (years)	14.4 ± 2.3	14.7 ± 2.3	14.0 ± 2.3	14.7 ± 2.3	14.5 ± 2.5	15.0 ± 1.9
Height (m)	1.6 ± 0.1	1.6 ± 0.1	1.7 ± 0.1	1.6 ± 0.1	1.6 ± 0.1	1.7 ± 0.1
Weight (kg)	96.1 ± 23.4 ^a^	92.2 ± 18.6 ^b,c,d^	101.2 ± 27.7	104.1 ± 23.9	101.6 ± 21.4	107.2 ± 26.6
BMI (kg/m^2^)	36.3 ± 6.7 ^a^	35.9 ± 5.8	36.7 ± 7.7	38.3 ± 6.9	38.3 ± 6.7	38.4 ± 7.2
Waist (cm)	109.7 ± 15.3 ^a^	106.2 ± 13.4 ^b,c,d^	114.9 ± 16.4	123.7 ± 17.8	121.5 ± 18.7	126.3 ± 16.8
WHR	0.9 ± 0.1 ^a^	0.9 ± 0.1 ^b,c,d^	1.0 ± 0.1	1.0 ± 0.1	1.0 ± 0.1	1.0 ± 0.1
Glucose (mg/dL)	86.3 ± 6.6 ^a^	85.4 ± 6.8 ^d^	87.7 ± 6.1	94.7 ± 39.1	97.5 ± 53.1	91.4 ± 8.3
Triglycerides (mg/dL)	96.0 ± 36.9 ^a^	94.5 ± 32.0 ^c,d^	98.2 ± 43.3 ^c,d^	158.7 ± 63.2	158.7 ± 53.4	158.8 ± 74.6
HDL-cholesterol (mg/dL)	46.0 ± 0.7 ^a^	46.5 ± 9.5 ^c,d^	45.3 ± 7.4 ^c,d^	38.7 ± 7.7	39.9 ± 7.0	37.3 ± 8.3
HR (bpm)	83.9 ± 12.9 ^a^	84.8 ± 12.8	82.6 ± 13.0 ^c^	87.1 ± 13.7	88.4 ± 11.7	85.4 ± 15.8
DBP (mmHg)	75.5 ± 5.8	75.8 ± 5.6	75.0 ± 6.2	80.5 ± 8.5	86.0 ± 5.5	75.8 ± 8.0
SBP (mmHg)	120.9 ± 10.1 ^a^	119.5 ± 8.8 ^c,d^	123.0 ± 11.5	130.9 ± 10.7	130.0 ± 11.5	131.9 ± 9.8

^a^: *p* < 0.05 vs. all obese subjects with metabolic syndrome; ^b^: *p* < 0.05 vs. obese males without metabolic syndrome; ^c^: *p* < 0.05 vs. obese females with metabolic syndrome; ^d^: *p* < 0.05 vs. obese males with metabolic syndrome.

**Table 2:** Effects of a three-week body weight reduction program on BMI (body mass index), OLSB (one-leg standing balance), SCT (stair climbing test) and FSS (fatigue severity score) in the recruited pediatric population, including obese subjects with or without metabolic syndrome, subdivided into females and males groups.

Parameter	Obese without Metabolic Syndrome			Obese with Metabolic Syndrome		
	All	Females	Males	All	Females	Males
BMI (kg/m^2^)						
Pre	36.3 ± 6.7 ^b^	35.9 ± 5.8	36.7 ± 7.7	38.3 ± 6.9	38.3 ± 6.7	38.4 ± 7.2
Post	34.8 ± 6.4 ^a,b^	34.5 ± 5.6 ^a^	35.1 ± 7.3 ^a^	36.7 ± 6.6 ^a^	36.8 ± 6.5 ^a^	36.6 ± 6.9 ^a^
FM (kg)						
Pre	43.7 ± 8.2	43.4 ± 7.2	44.3 ± 9.9	41.4 ± 12.0	43.2 ± 4.5	40.1 ± 16.3
Post	40.8 ± 7.2 ^a^	41.7 ± 6.8 ^a^	39.4 ± 8.0 ^a^	39.0 ± 10.7 ^a^	41.2 ± 4.3	37.3 ± 14.4
FM (%)						
Pre	43.8 ± 5.5	45.1 ± 5.1 ^c^	42.1 ± 5.5 ^e^	43.9 ± 6.1	46.1 ± 5.1 ^d^	41.2 ± 6.1
Post	42.7 ± 5.8 ^a^	44.3 ± 5.3 ^a,c,d^	40.7 ± 5.9 ^a,e^	43.0 ± 5.0 ^a^	45.2 ± 5.1 ^a,d^	40.3 ± 5.5 ^a^
FFM (kg)						
Pre	54.3 ± 9.5					
Post	53.6 ± 9.9	48.0 ± 6.4 ^c^	63.3 ± 6.8	54.1 ± 7.2	49.2 ± 3.5 ^d^	58.1 ± 7.1
OLSB_L_ (s)						
Pre	45.6 ± 19.2	47.4 ± 18.2	43.4 ± 20.2	42.8 ± 21.1	43.0 ± 21.0	42.6 ± 21.4
Post	51.4 ± 15.1 ^a^	52.8 ± 14.0 ^a^	49.5 ± 16.4 ^a^	49.1 ± 18.0 ^a^	49.1 ± 18.7 ^a^	49.2 ± 17.2 ^a^
OLSB_R_ (s)						
Pre	45.2 ± 19.3	47.5 ± 18.4	42.3 ± 20.1	43.7 ± 21.0	46.3 ± 20.3	40.6 ± 21.7
Post	51.8 ± 15.2 ^a^	53.7 ± 13.4 ^a^	49.3 ± 17.0 ^a^	48.4 ± 17.8 ^a^	50.3 ± 17.3 ^a^	46.1 ± 18.2 ^a^
SCT (s)						
Pre	3.1 ± 0.6	3.1 ± 0.5 ^c,d^	3.0 ± 0.7 ^e^	3.1 ± 0.5	3.3 ± 0.6 ^d^	2.9 ± 0.4
Post	2.8 ± 0.5 ^a^	2.9 ± 0.4 ^a^	2.8 ± 0.5 ^a,e^	2.9 ± 0.5 ^a^	3.0 ± 0.6 ^a,d^	2.7 ± 0.4 ^a^
FSS						
Pre	26.0 ± 6.4	23.8 ± 5.0	29.7 ± 7.1 ^a^	27.3 ± 10.2	28.5 ± 9.5	26.4 ± 11.8
Post	22.7 ± 6.4 ^a^	20.5 ± 4.4 ^a^	26.2±7.7 ^a^	20.6 ± 9.2 ^a^	20.3 ± 9.2 ^a^	20.8 ± 10.3 ^a^

^a^: *p* < 0.01 vs. the corresponding post value; ^b^: *p* < 0.01 vs. all obese subjects with metabolic syndrome; ^c^: *p* < 0.01 vs. obese males without metabolic syndrome; ^d^: *p* < 0.01 vs. obese males with metabolic syndrome; ^e^: *p* < 0.01 vs. obese females with metabolic syndrome.

## Abbreviations

BMI, body mass index; BWRP, body weight reduction program; DBP, diastolic blood pressure; F, female; FFM, fat-free mass; FM, fat mass; FPG, fasting plasma glucose; FSS, fatigue severity scale; HDL, high-density lipoprotein; HR, heart rate; IDF, International Diabetes Federation; L, left; LLMP, lower limb muscle power; M, male; METS, metabolic syndrome; OB, obese; OLSB, one-leg standing balance; R, right; SBP, systolic blood pressure; SCT, stair climbing test; SD, standard deviation; WC, waist circumference; WHR, waist to hip ratio.
